# Solvent and Water Mediated Structural Variations in Deoxynivalenol and Their Potential Implications on the Disruption of Ribosomal Function

**DOI:** 10.3389/fmicb.2016.01239

**Published:** 2016-08-17

**Authors:** Nora A. Foroud, Roxanne A. Shank, Douglas Kiss, François Eudes, Paul Hazendonk

**Affiliations:** ^1^Lethbridge Research and Development Centre, Agriculture and Agri-Food CanadaLethbridge, AB, Canada; ^2^Department of Chemistry and Biochemistry, University of LethbridgeLethbridge, AB, Canada

**Keywords:** deoxynivalenol (DON), fusarium head blight (FHB), fusarium graminearum, NMR spectroscopy, mycotoxins, chemical structure

## Abstract

Fusarium head blight (FHB) is a disease of cereal crops caused by trichothecene producing *Fusarium* species. Trichothecenes, macrocylicic fungal metabolites composed of three fused rings (A–C) with one epoxide functionality, are a class of mycotoxins known to inhibit protein synthesis in eukaryotic ribosomes. These toxins accumulate in the kernels of infected plants rendering them unsuitable for human and animal consumption. Among the four classes of trichothecenes (A–D) A and B are associated with FHB, where the type B trichothecene deoxynivalenol (DON) is most relevant. While it is known that these toxins inhibit protein synthesis by disrupting peptidyl transferase activity, the exact mechanism of this inhibition is poorly understood. The three-dimensional structures and H-bonding behavior of DON were evaluated using one- and two-dimensional nuclear magnetic resonance (NMR) spectroscopy techniques. Comparisons of the NMR structure presented here with the recently reported crystal structure of DON bound in the yeast ribosome reveal insights into the possible toxicity mechanism of this compound. The work described herein identifies a water binding pocket in the core structure of DON, where the 3_OH_ plays an important role in this interaction. These results provide preliminary insights into how substitution at C_3_ reduces trichothecene toxicity. Further investigations along these lines will provide opportunities to develop trichothecene remediation strategies based on the disruption of water binding interactions with 3_OH_.

## Introduction

Deoxynivalenol (DON) belongs to a class mycotoxins called trichothecenes and are produced by *Fusarium* species involved cereal crop diseases, such as Fusarium head blight (FHB; Foroud and Eudes, 2009). The responsible fungal species infect wheat and other small grains during flowering and kernel development stages and mycotoxins accumulate in the kernels of infected plants (Foroud et al., 2014). Ingestion of trichothecene-contaminated grain is harmful for human and animal consumers (Pestka, 2010). Trichothecenes are known to induce programmed cell death (apoptosis) by exerting ribotoxic effects on eukaryotic cells (Shifrin and Anderson, 1999; Rocha et al., 2005). Interestingly, like many antibiotics, trichothecenes interfere with ribosome function, and act as potent inhibitors of protein synthesis in eukaryotes (Ueno et al., 1968; McLaughlin et al., 1977). Earlier it was hypothesized that trichothecenes make direct contact with the ribosomal protein RPL3 (Gilly et al., 1985). Moreover, three domains of RPL3 function as a “rocker switch” that dynamically coordinates amino acyl-tRNA (aa-tRNA) and ribosome during translation elongation (Meskauskas and Dinman, 2008)—thus, an interaction of these toxins with RPL3 would result in an inhibition of protein synthesis. This hypothesis was validated in yeast where W225C or W225R mutations in the highly conserved W-finger of RPL3 conferred toxin resistance (Mitterbauer et al., 2004). The close proximity of RPL3 with the peptidyl transferase center (PTC) suggests that trichothecenes interfere directly with peptidyl transferase activity (Mitterbauer et al., 2004). Recent x-ray crystallography studies of toxin-bound yeast ribosomes, clearly shows trichothecene (DON, T-2 toxin and verrucarin A) binding to the A-site of the PTC (Garreau De Loubresse et al., 2014), which would impair peptide bond formation during translation elongation.

The most effective method to minimize trichothecene contamination of food/feed grain is to grow cultivars with FHB resistance and to employ strategic disease management practices, such as those previously described (Dill-Macky and Jones, 2000; Krupinsky et al., 2002; McMullen et al., 2008, 2012; Foroud et al., 2014). A major challenge is that “immunity” to FHB has not been identified in cultivated cereals, and the availability of highly resistant cultivars is limiting since resistance tends to be associated with poor agronomics (Foroud et al., 2014). Ongoing efforts have led to some improvements over the years (for an overview see McMullen et al., 2012, and other publications in the current issue of Frontiers in Microbiology), meanwhile FHB continues to have significant impact. Furthermore, no remediation strategies are available for detoxification or sequestration of trichothecenes. That being said, grain cleaning strategies can be employed to remove some of the contaminated roughage from the grain (Tittlemier et al., 2014) and biological mechanisms to detoxify trichothecenes have been identified (Fuchs et al., 2002; Poppenberger et al., 2003; Boutigny et al., 2008).

**Scheme 1:**
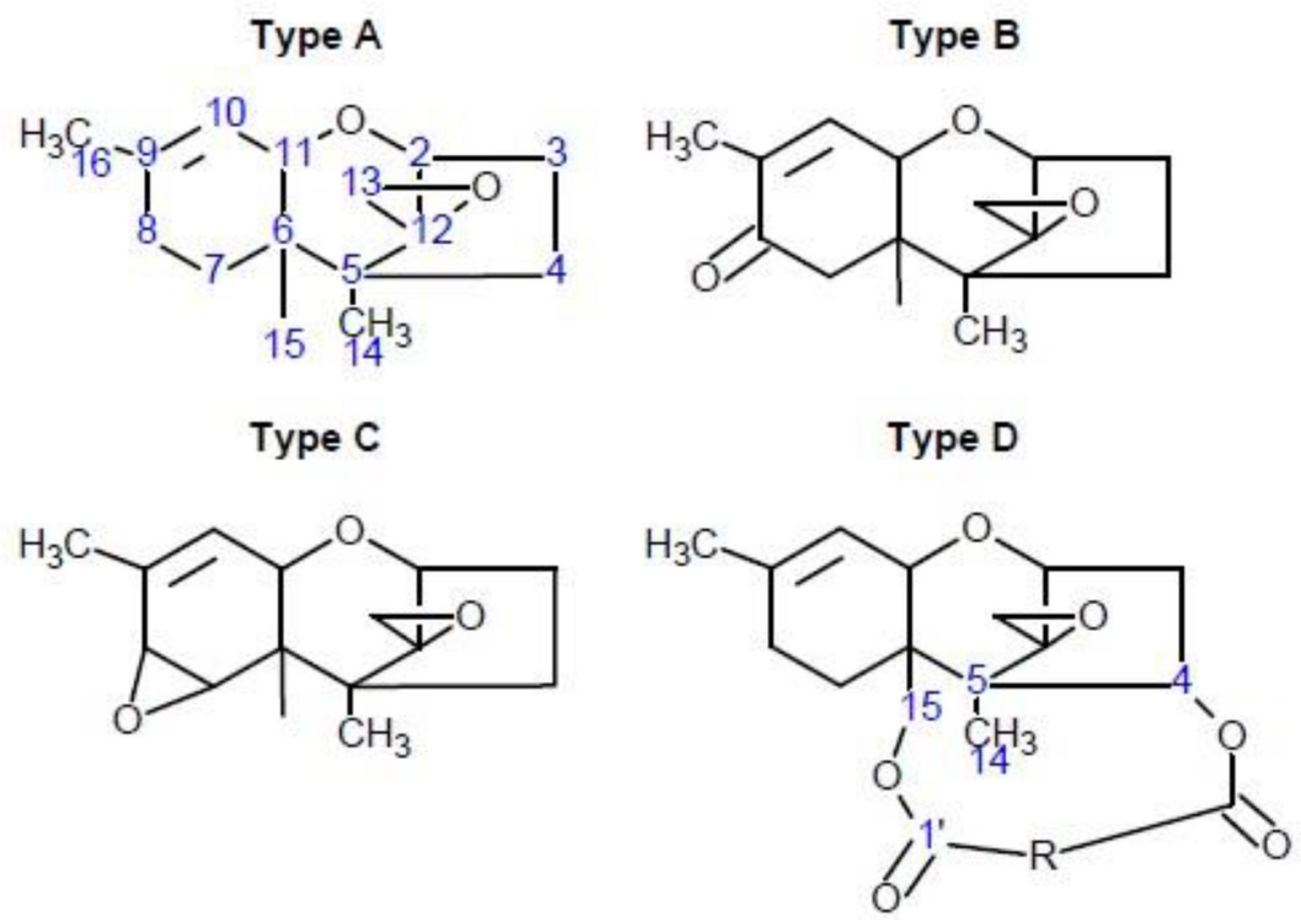
**The general core structures for types A–D trichothecenes**. The core carbons are numbered for type A, and the substituent carbons for the macrocyclic type D trichothecene structure are also numbered for clarity.

Trichothecenes are composed of three fused rings: the cyclohexene (A-ring) is fused to the tetrahydropyran (B-ring), which is bridged by a 2-carbon chain at C_2_ and C_5_ thereby forming a cyclopentyl moiety (C-ring). In addition, an epoxide functionality is attached at C_12_ which is common to the B- and C-rings (Scheme 1) (Cole and Cox, 1981). Side chains at C_3_, C_4_, C_7_, C_8_, and C_15_ are variable, although primarily consist of H, OH, or OC(= O)CH_3_. Trichothecenes fall into four classes (types A–D) (McCormick et al., 2011), where either A and B are produced by *Fusarium* species. DON is a type B trichothecene, which is characterized by a ketone at C_8_, and has hydroxyl groups at C_3_, C_7_, and C_15_. The epoxide ring is essential for toxicity (Ehrlich and Daigle, 1987). which are unusually stable in the trichothecenes (Pronyk et al., 2006; Bullerman and Bianchini, 2007). Some bacterial species can open the epoxide ring, forming de-epoxynivalenol (DOM-1) (Fuchs et al., 2002; Schatzmayr et al., 2006). No de-epoxy trichothecenes have been reported in plants infected with trichothecene-producing *Fusarium* species. Other modifications, *in planta*, that show reduced toxicity include DON-thiol, and -glucoside conjugates (Gardiner et al., 2010; Kluger et al., 2013; Stanic et al., 2015). The former were observed at C_9_, disrupting the double bond in the A-ring (Kluger et al., 2013), and at C_8_, disrupting the R5 keto functional group at Gardiner et al. (2010). The latter are catalyzed by plant UDP-glucostranferases that yield DON-3-glucosides (Poppenberger et al., 2003). Generally, modifications at C_3_ reduces toxicity; for example, acetylation, produces 3-*O*-acetyl-DON which is significantly less toxic than DON (Desjardins et al., 2007; McCormick, 2009).

Structural knowledge of trichothecenes has driven detoxification/remediation strategies. Trichothecenes from all classes have been studied primarily in chloroform (Cole and Cox, 1981; Savard et al., 1987; Greenhalgh et al., 1989), which obviously does not emulate their biological environment. A single report on the effect of solvent DON and NIV structures provides compelling evidence for significant structural variation in different chemical environments. Jarvis et al. (1990) provided preliminary evidence for a second minor configuration in a single crystal X-ray diffraction (SCXRD) study of NIV recrystallized from a mixture of methanol-d_4_ (CD_3_OD) and water. The authors compared changes in ^13^C nuclear magnetic resonance (NMR) spectra of both DON and NIV in CDCl_3_ with those in acetone-d_6_, CD_3_OD, and DMSO-d_6_, and proposed that the second configuration in both toxins is an isomer resulting from a rearrangement of the ketone at C_8_ and hydroxyl at C_15_ to a hemiketal linkage between C_8_ to C_15_.

Historically, CDCl_3_ was the solvent of choice as most trichothecenes are soluble in it, and it seems to minimize problems associated with aggregation which is at the heart of their poor solubility in water. Chloroform is a significant departure from the cellular environment. Although the cytosol is primarily aqueous, some hydrophobic environments with limited free water exist. As such, trichothecene structure and dynamics should be observed in both types of environments, where the presence of free water is controlled to gain a better understanding of how these toxins interact with and move through the cell.

A more comprehensive structural analyses and a dynamic understanding of the trichothecene-ribosome interaction and inhibition mechanisms will lead to more advancements in this area. With some exceptions, the majority of the structural analysis of trichothecenes reported to date was carried out 20–30 years ago, and often with the purpose of basic structural determination and identification. Technological advances in structural determination have made it possible to perform more detailed structural analysis and dynamics. A small number of dynamic studies have been reported among the different classes of trichothecenes (Jarvis et al., 1990; Fragaki et al., 2008; Steinmetz et al., 2008; Chaudhary et al., 2011; Shank et al., 2011). Density functional theory (DFT) computations were carried out by Nagy et al. (2005), on the internal dynamics of DON, which suggested that the lowest energy configuration contains an internal H-bonding network. The proposed H-bonding occurs between the C_8_ carbonyl oxygen and the C_7_ hydroxyl hydrogen, which in turn is linked to the hydroxyl hydrogen at C_15_. The energy of this interaction is significantly lower than all other locally optimal configurations considered; however, H-bonding and bridging with water was not taken into account in this study. Also recent evidence presented by our group on water binding in T-2 toxin (Chaudhary et al., 2011) demonstrates that the H-bonding networks, both intra- and inter-molecular, of all the trichothecenes should be carefully considered.

The current study was designed to determine whether different solvent properties induce structural changes in DON, where the presence of free and bound water is controlled. High resolution ^1^H and ^13^C solution state spectra are presented which provides novel insights into the structure and dynamics of DON in different chemical environments. The structural implications on the toxicity mechanisms and biological interactions of DON are discussed.

## Materials and methods

### Materials

4-Deoxynivalenol was purchased from Sigma, (DON; CAS 51481-10-8), with estimated purity >95%. The delivered sample, which had a flaky appearance, was dried under dynamic vacuum to remove residual water.

NMR samples of DON were prepared in CDCl_3_ to a concentration of 1 mg mL^−1^ with TMS as an internal reference for both ^13^C and ^1^H. CDCl_3_ was dried over molecular sieves to prevent further introduction of water through the solvent. A sample was also prepared in dried DMSO-d_6_; however for the samples in acetone-d_6_, THF-d_4_ and methanol-d_4_ the solvents were not dried. All deuterated solvents were purchased from Sigma-Aldrich.

### ^1^H solution-state NMR experiments

All NMR spectra were acquired using a Bruker Avance 2 300 MHz spectrometer, outfitted with a 5 mm HX PABBO BB probe. The magnetic field strength is 7.05 Tesla, with larmor frequencies of 300.13 MHz for ^1^H. All experiments were performed at ambient temperature (25°C). Typical 1D ^1^H spectra were recorded as 128 transients, using a 90° pulse width of 12.4 μs, and a recycle delay of 1.5 s. Similarly typical 1D ^13^C spectra were obtained in 4000 transients, using a 90° pulse width of 7.6 μs, and a recycle delay of 4.0 s, as 64 K points over a spectral window of 18 kHz.

The homonuclear magnitude gradient ^1^H COSY spectrum was acquired in 256 increments over a spectral width of 1800 Hz (12.0 ppm) in both dimensions, using a recycle delay of 1.5 s. 64 transients were collected for each increment, having 4096 points. The direct and indirect dimensions have a digital resolution of 0.88 and 7.03 Hz, respectively, before zero filling.

The gradient ^1^H NOESY spectrum was acquired in at least 256 increments covering a spectral width of 1802.45 Hz (12.0 ppm) in both dimensions, using a recycle delay of 20 s and an array of mixing times of 0.5, 1.0, 2, 5, 7, and 10 s. Eight transients were collected for each increment, having 4096 points. The direct and indirect dimensions have a digital resolution of 0.88 and 7.03 Hz, respectively, before zero filling. Inversion recovery experiments determined that the T_1_s of all DON and water signals were below 3 s.

The ^1^H-^13^C HSQC spectrum was acquired in 128 increments, using a recycle delay of 2.0 s, and a spectral width of 4006.41 Hz (13.34 ppm) in the direct dimension and 12,500 Hz (165.62 ppm) in the indirect dimension. 152 transients were collected for each increment, having 1024 points, which was zero filled to 4096 points. The *t*_1_ dimension was linear forward predicted to 256 points and further zero filled to 1024 points. The direct and indirect dimensions have a digital resolution of 3.9 and 98 Hz, respectively.

The ^1^H-^13^C HMBC spectrum was acquired in 256 increments using a recycle delay of 2.0 s, and a spectral width covering 1951.60 Hz (6.50 ppm) in the direct dimension and 14,268 Hz (190.24 ppm) in the indirect dimension. One-hundred transients were collected for each increment, having 1024 points and the FID was zero-filled up to a value of 4096 points. The *t*_1_ dimension was linear forward predicted to 512 points and further zero filled to 1024 points. The direct and indirect dimensions have a digital resolution of 0.52 and 56 Hz, respectively.

Drying experiments were performed at ambient temperature by sequential addition of individual molecular sieves to the NMR tube between consecutive NMR measurements, over the course of 3 h where the water to DON ratio was monitored over a period of 72 h.

### Simulations

The SpinWorks processing and simulation software suite, developed by Marat (2009) at the University of Manitoba, was used to simulate the data obtained for the 300 MHz ^1^H spectra. The FIDs were zero-filled to 256 K points and were subjected to Gauss-Lorentz apodization with a line broadening between –1.00 to –0.50 Hz, and a Gaussian broadening of 0.33, depending on the signal-to-noise ratio in the data. The spectra were simulated in two parts as the whole spin system could not be simulated at once. The sub-spectrum of the six-membered ring was simulated as a 10-spin ABC_3_DEFG system using 7_OH_, 7_β_, 10, 11, 15_α_, 15_β_, 15_OH_, 16. Similarly, the sub-spectrum associated with the remaining hydrogens: 2, 3, 3_OH_, 4_α_, 4_β_, 14, 13_α_, and 13_β_ was simulated as a ten-spin ABCDEF_3_GH spin system. Long range couplings were considered up to five bonds, and an inherent line-width of 0.3 Hz along with Gaussian line shapes was used to fit the data. In the A-ring simulation typically 2000 transitions were assigned to within RMS deviations below 0.03 Hz, and a largest absolute differences of less than 0.08 hz Standard deviations (SD) in all the spectral parameters ranged from 0.1000 to 0.001 hz In the C ring simulation, typically 2200 transitions were assigned with an RMS deviations below 0.03 Hz, and largest absolute differences less than 0.09 hz SD in all the spectral parameters range from 0.1000 to 0.001 Hz.

### Structural analysis

All proposed structures were calculated and optimized using ACD ChemSketch, from which internuclear distances were estimated for comparison to those obtained from the NOESY spectra in CDCl_3_, and DMSO-d_6_. All NOESY spectra were analyzed using Mestre NOVA software, using an LB of 3 Hz in each dimension. Integration of all the cross-peaks (CP) and auto-correlation peaks (ACP) was performed from which the CP to ACP ratio were computed and the cross-relaxation rate, *s*, was obtained using:

(1) $$\begin{array}{l} \text{tan}{\text{h}}^{-1}\left(\frac{{\text{I}}_{\text{CP}}}{{\text{I}}_{\text{ACP}}}\right)=\sigma \tau_{\text{mix}} \end{array}$$

The cross-relaxation rates were used to determine the internuclear distances, using 4_α_ and 4_β_ as well as 13_α_ and 13_β_ as references with internuclear distances of 181 and 186 pm respectively. The following relationship was used:

(2) $$\begin{array}{l} r_{ij}=r_{ref}\sqrt[{6}]{\frac{\sigma_{ref}}{\sigma_{ij}}} \end{array}$$

The EXSY CP were used to compute exchange rates between water and OH signals using:

(3) $$\begin{array}{l} \text{tan}{\text{h}}^{-1}\left(\frac{{\text{I}}_{\text{CP}}}{{\text{I}}_{\text{ACP}}}\right)=k\tau_{\text{mix}} \end{array}$$

## Results

### Full spectral assignment of DON in chloroform

The assignment of the ^1^H spectrum in CDCl_3_ differed significantly from those in previous reports on the basis of spectral simulation and new evidence from the COSY and NOESY spectra. In addition to the revision of the methylene proton assignments, accurate chemical shifts of the hydroxyl protons are offered here.

**Figure 1:**
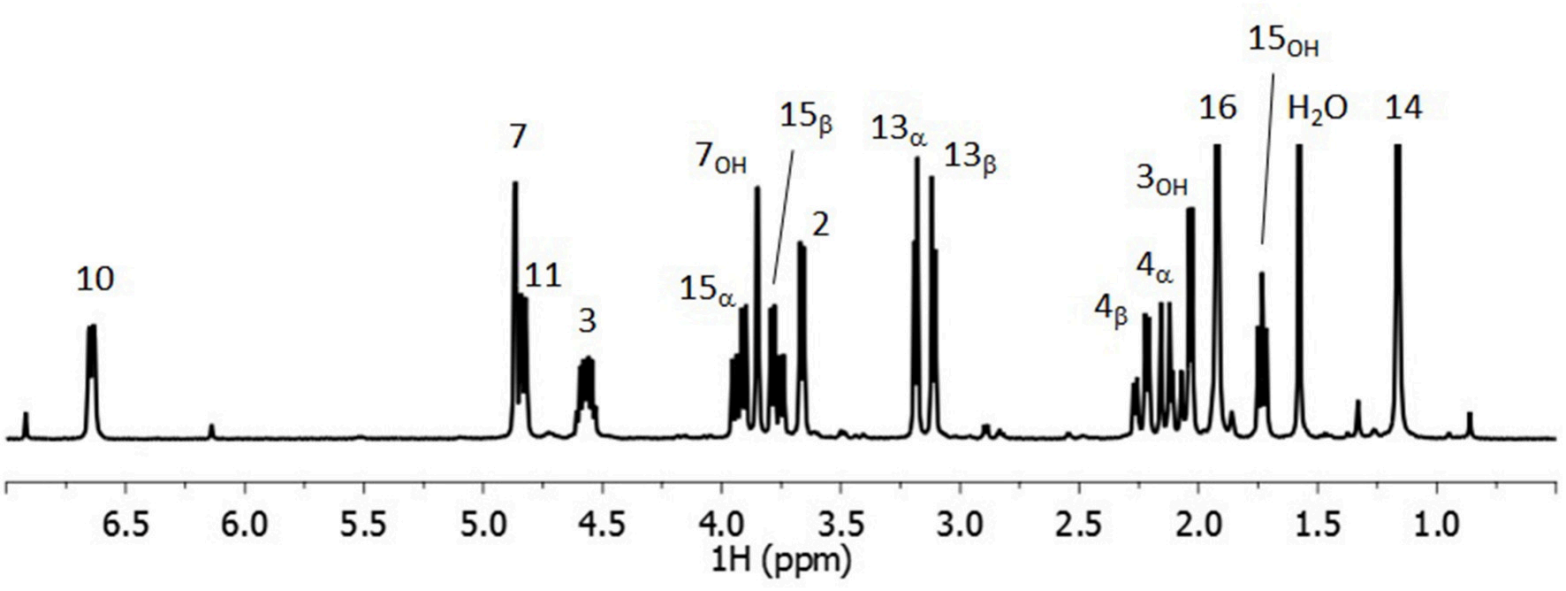
**300 MHz ^**1**^H of DON in dried CDCl_**3**_**.

**Figure 2:**
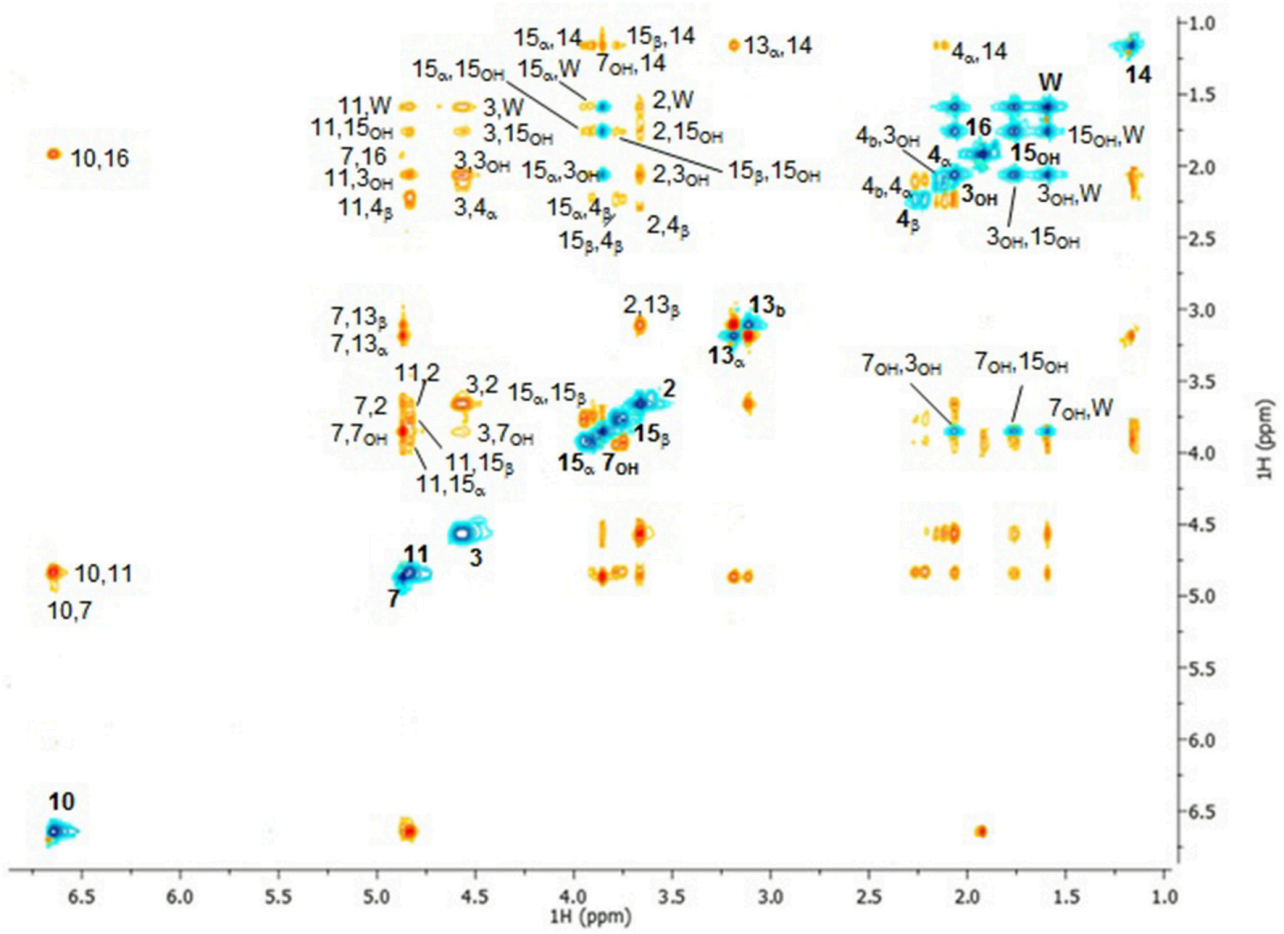
**300 MHz ^**1**^H NOESY of DON in CDCl_**3**_ with a mixing time of 3 s**.

The ^1^H spectrum of DON in CDCl_3_ is shown in Figure 1 in which the labeling convention of Savard et al. (1987) was employed as shown in Scheme 1. Where significant π-electron delocalization is expected, coupling constants of up to five-bonds were considered. The assignment of the ^1^H spectrum is given in Table 1 where the chemical shifts and coupling constants given alongside the diagnostic homonuclear CP from the COSY and the NOESY spectra. Figure 2 contains the NOESY spectrum of DON in CDCl_3_ measured using a mixing time of 3 s. The most prominent CP are labeled, and used to compute exchange rates and internuclear distances, which are shown in Table 2. The heteronuclear 2D experiments are not shown. The assignment corresponds closely to previous reports (Savard et al., 1987; Nagy et al., 2005) with some notable exceptions described below.

**Table 1:** **Chemical shifts (ppm) for DON in CDCl_**3**_ compared with literature**.

**^1^H**	**Exp**.	**Lit.^a^**	**COSY^b^**	**NOESY^d^**
2	3.671	3.62	3, 4_α_,11,14,	3
3	4.577	4.53	2, 3_OH_, 4_αβ_	2, 4_αβ_
3_OH_	2.066	N.O.^c^	3	$15_{\text{OH}}$, W
4_α_	2.129	2.21	2, 3, 4_β_,11	3, 4_β_,14
4_β_	2.247	2.07	3, 4_α_,11,13_β_,14	2, $3_{\text{OH}}$, 4_α_,11, $15_{\alpha \beta }$
7	4.876	4.83	7_OH_	7_OH_
7_OH_	3.861	N.O.	7	7,14,W
10	6.652	6.61	11,16	11,16
11	4.843	4.80	2,4_αβ_,10,15_αβ_,16	2,$3_{\text{OH}}$,$4_{\beta }$,10,$15_{\alpha \beta \text{OH}}$,16
13_α_	3.195	3.07	13_β_	13_β_,14
13_β_	3.121	3.15	13_α_,4_β_	2,13_α_
14	1.170	1.13	2, 4_β_	$4_{\alpha }$, $7_{\text{OH}}$,$15_{\alpha }$
15_α_	3.932	3.73	15_β_,15_OH_,11	$4_{\beta }$,14,15_β_,15_OH_
15_β_	3.778	3.89	15_α_,15_OH_,11	$4_{\beta }$,11, 14,15_α_,15_OH_
15_OH_	1.759	N.O.	15_α_,15_β_	$3_{\text{OH}}$,15_αβ_,W
16	1.930	1.86	10,11	10

a *Savard and Blackwell (1994)*.

b *EXSY crosspeaks are in blue*.

c *Not observed*.

d *NOESY crosspeaks of note are in red*.

**Table 2:** **Internuclear distances calculated from NOESY spectrum in CDCl_**3**_, at a mixing time of 3 s, compared with computed boat and chair geometries**.

***N*^a^_i_**	***N_j_***	***r*_ij_^b^**	***r*_ij_(boat)^c^**		***r*_ij_(chair)^c^**	
2	3	2.06	2.34	0.08^d^	2.36	0.09^d^
2	7	3.97	**5.20**	**1.52**	3.92	0.00
2	13_β_	2.30	2.50	0.04	2.53	0.05
2	15_α_	3.70	4.44	0.55	**5.31**	**2.59**
2	3_OH_	2.56	2.87	0.09	2.74	0.03
2	4_β_	3.68	4.23	0.30	4.26	0.34
2	W	**2.99**				
3	3_OH_	2.11	**2.75**	**0.41**	2.24	0.02
3	4α	1.77	2.25	0.24	2.29	0.27
3	4_β_	3.06	2.91	0.02	2.91	0.02
3	W	**2.84**				
7	10	3.72	4.13	0.17	4.14	0.17
7	13_α_	2.11	2.20	0.01	1.76	0.12
7	13_β_	2.42	**3.92**	**2.25**	2.39	0.00
7	7_OH_	2.21	2.20	0.00	2.28	0.00
10	11	2.09	**2.90**	**0.65**	2.54	0.20
10	16	3.10	2.96	0.02	3.03	0.01
11	2	2.91	**4.16**	**1.57**	3.57	0.44
11	15_α_	2.70	2.65	0.00	2.81	0.01
11	15_β_	2.44	2.35	0.01	2.61	0.03
11	3_OH_	2.53	2.79	0.07	2.37	0.02
11	4_β_	1.97	**4.13**	**4.65**	2.46	0.24
11	W	**3.14**				
14	13_α_	2.87	3.04	0.03	3.10	0.05
14	7_OH_	2.98	3.67	0.48	3.58	0.37
13_α_	13_β_	1.76	1.87	0.01	1.88	0.01
15_α_	15_β_	2.00	1.81	0.03	1.80	0.04
15_α_	15_OH_	2.46	2.24	0.05	2.71	0.06
15_α_	4_β_	2.35	1.89	0.21	1.91	0.20
15_α_	W	**2.93**				
15_β_	15_OH_	2.59	2.29	0.09	2.78	0.04
15_β_	4_β_	2.98	3.32	0.11	3.23	0.06
15_β_	W	**3.22**				
3_OH_	4_β_	2.45	2.91	0.22	**3.37**	**0.84**
4α	14	2.48	2.94	0.22	2.99	0.26
4α	4_β_	1.81	1.82	0.00	1.82	0.00
7_OH_	13_α_	2.49	**4.36**	**3.52**	2.60	0.01
7_OH_	15_β_	3.58	3.46	0.02	3.95	0.14
			χ^2e^	17.64		6.73
			(σ)^e^	(0.74)		(0.46)

a *Hydrogen nucleus*.

b *Internuclear distance in Å computed from NOESY crosspeaks using Equations (1) and (2) (green indicates distances to the bound water molecule)*.

c *Predicted estimates for internuclear distances using AM1 computations*.

d *Mean deviation squared*.

e *Chi squared based on 33 distances in Å^2^, and s is the standard deviation in Å (red and blue highlights the largest contributor to the chi squared of boat and chair conformations, respectively)*.

**Figure 3:**
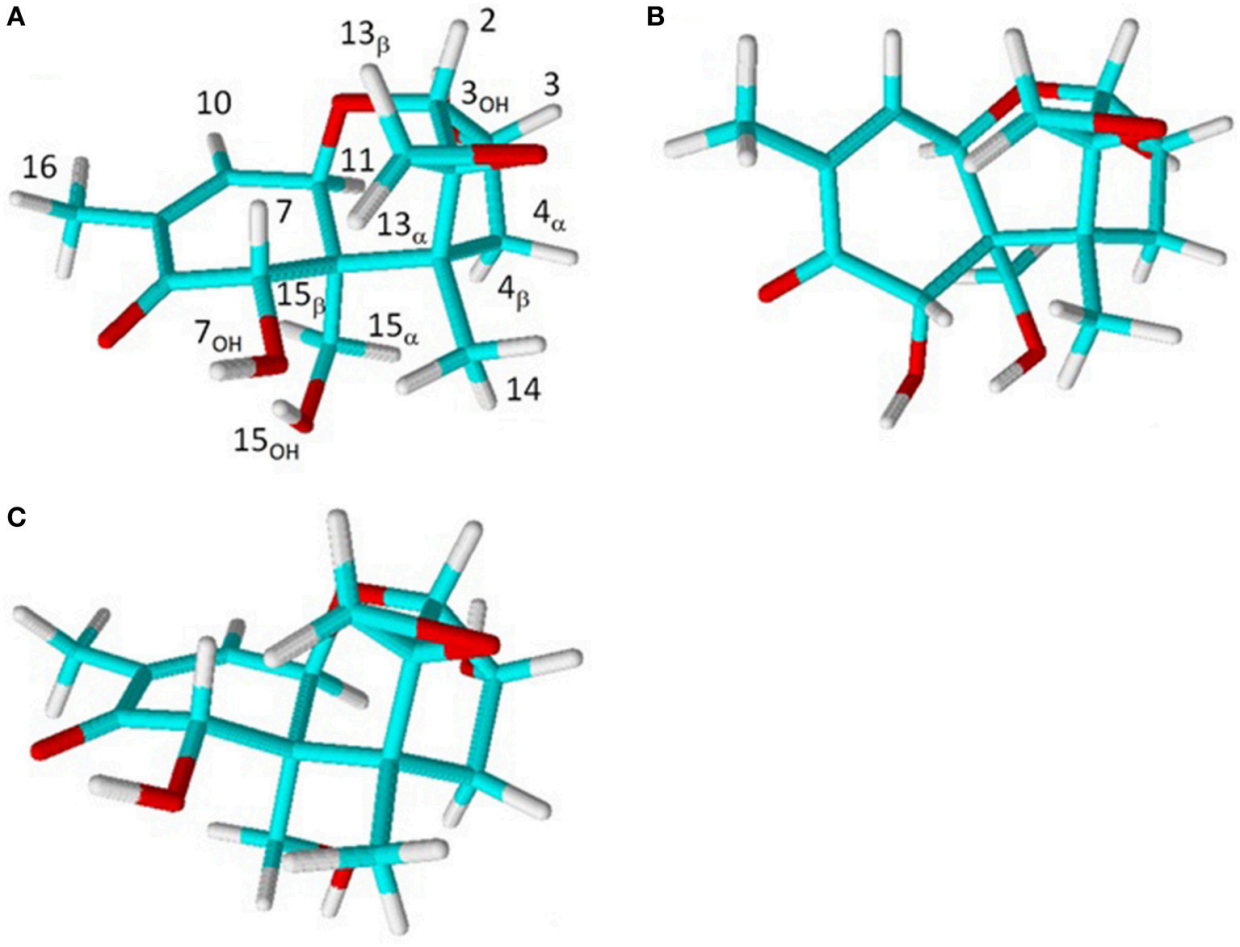
**Predicted structure of DON in (A) CDCl_**3**_ chair, (B) CDCl_**3**_ boat, and (C) DMSO-d_**6**_ chair B-ring conformations**.

The assignment of 4_α_and 4_β_is now reversed, where the chemical shift of the former is smaller than the latter (Table 1). This was confirmed by NOESY, where a strong cross-peak between 4_β_ and 11 is observed and that between 4_β_ and 14 is weak, which stands in contrast to the strong cross-peak 4_α_ and 14 and that between 4_α_ and 11 being weak. Furthermore, the cross-peak between 3 and 4_β_ is much stronger than the 1 to 4_α_, and those from both 15_α_ and 15_β_ to 4_β_ are much stronger than those to 4_α_. This reassignment clearly makes it easier to appreciate the relative order in the chemical shifts where 4_β_ is larger than 4_α_, which is consistent with 4_β_ being eclipsed by the hydroxyl oxygen on C_3_ (see Figure 3A). Correspondingly 4_α_ eclipses 3 as indicated by the vicinal coupling of 10.98 Hz, which could be easily confused with a *trans* vicinal coupling causing 4_α_ and 4_β_ to be interchanged.

The assignments of the protons on the epoxy ring were reversed with respect to previous reports (Savard and Blackwell, 1994), where 13_α_, at 3.195 ppm, has a significant cross-peak with 14, while 13_β_, at 3.121 ppm, has a prominent cross-peak with 2. Additionally, the larger cross-peak between 7 and 13_α_ compared with 7 and 13_β_ further supports this assignment. Hence, the larger chemical shift of 13_α_ compared with 13_β_is due to its proximity to 15_OH_.

The methylene protons 15_α_ and 15_β_ are not easy to assign; however, NOESY CP provide the most convincing evidence that 15_α_ is closer to 14, while 15_β_ is closer to 11. Thus, 15_α_ nearly eclipses C_5_, while similarly 15_β_ is almost eclipses C_11_ and thus is oriented underneath the B ring, placing 15_OH_ in the correct orientation to undergo H-bonding to 7_OH_. The larger chemical shift of 15_α_ can be ascribed to its closer proximity to 7_OH_. This indicates a preference for the gauche (with a dihedral angle C_7_-C_6_-C_15_-O_15_ = 330°) rotamer, which is stabilized by transient H-bonding to 7_OH_.

**Figure 4:**
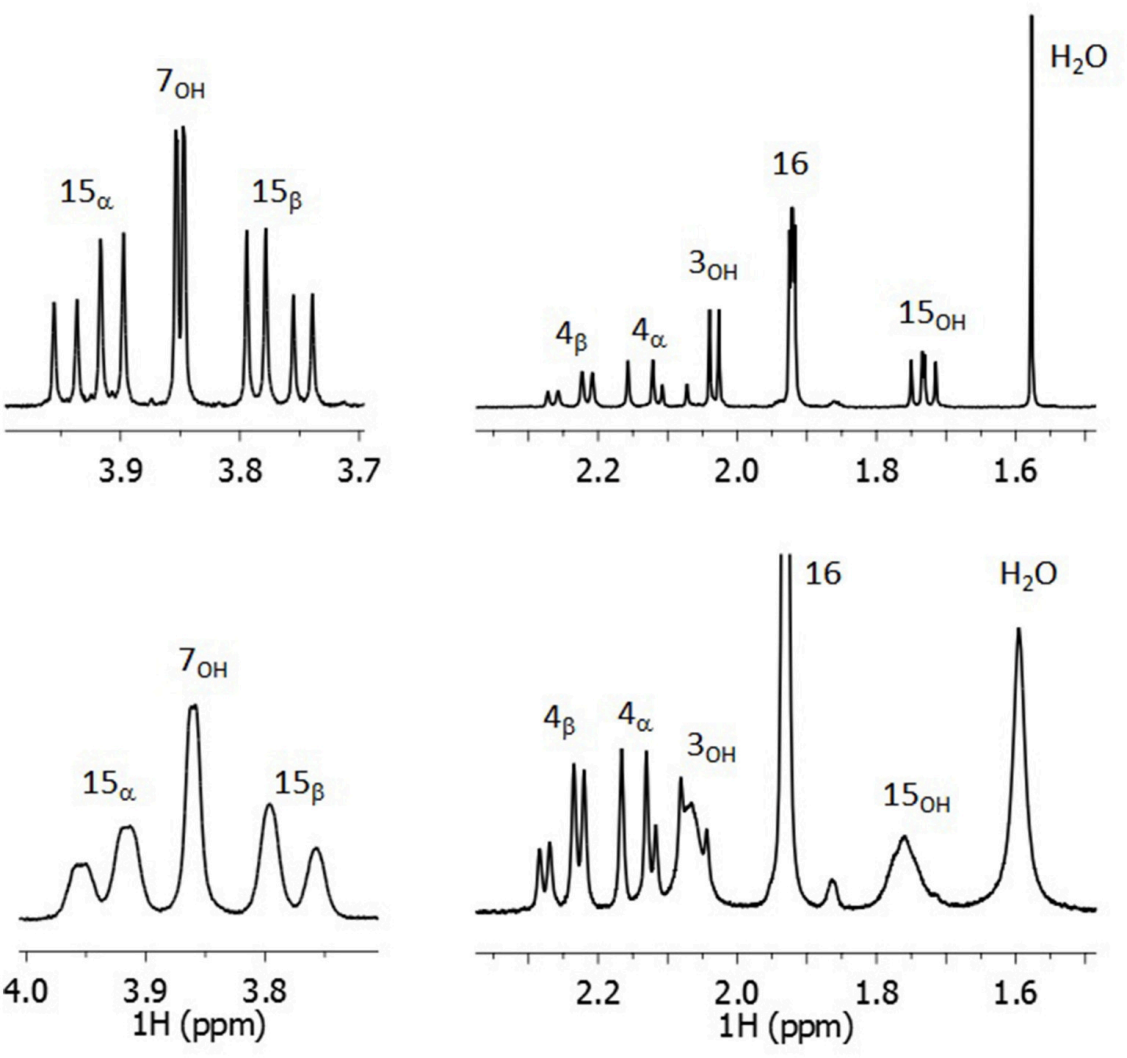
**Expansions of OH regions of the 300 MHz ^**1**^H spectrum of DON in dried (Top) and wet (Bottom) CDCl_**3**_**.

The shifts and couplings involving of the hydroxyl protons went previously unreported presumably due to being obscured by chemical exchange broadening with excess free water in the solvent. To illustrate the loss of resolution in those signals, Figure 4 compares the spectra for DON prepared with CDCl_3_ that was either dried or not dried. The high resolution achieved in the OH signals of the dried sample indicates that very slow exchange takes place with water. Line broadening in the wet sample suggests faster exchange; however, 7_OH_ remains relatively narrow. The EXSY cross-peak intensities give exchange rates in the dry sample for 7_OH_ is 0.04 s^−1^ while those for 15_OH_ and 3_OH_ are 0.12 s^−1^ suggesting that strong H-bonding between 7_OH_ and the carbonyl oxygen hinders water exchange.

The natural line width of the water and OH signal in the dry sample are below 0.2 Hz as their T_2_s occur near 1.5 s. Consequently, the effect of chemical exchange on the OH chemical shifts is negligible and thus they reflect the true shift of the OH environment. The OH shifts in the dry sample support preferential H-bonding to 7_OH_ as its shift is much larger than those of 15_OH_ and 3_OH_.

The water peak contains only one component in approximately equal stoichiometric proportions to DON. The signal is extremely narrow lacking any significant fine structure, indicating that it has significant mobility. In an attempt to remove water bound to DON, a sample was prepared in dried solvent where molecular sieves were added over a period of 72 h. There was an initial slight decrease in the ratio of the water to DON, after which, the ratio remained constant with time, and the number of sieves added. The stoichiometric ratio of water to DON converged to 1:1, meaning that one water molecule is very strongly bound to each DON molecule.

The ^13^C signals were assigned using HSQC and HMBC. Table 3 shows the ^13^C assignment for CDCl_3_ along with all the diagnostic heteronuclear CP. All the ^13^C chemical shifts are summarized in Table 4 and are reported with respect to TMS in all 5 solvents. The ^13^C chemical shift assignment in CDCl_3_ is the same as previously reported. Additional structural observations can be made from when they are compared between the different solvents.

**Table 3:** **The assignment of the carbon chemical shifts and observed ^**1**^H-^**13**^C correlations for DON in CDCl_**3**_**.

**^13^C**	**δ (ppm)^a^**	**HSQC**	**HMBC**
2	80.80	2	11
3	69.17	3	2,3_OH_,4_αβ_
4	43.21	4_α_, 4_β_	2,4_β_,3_OH_
5	46.46		14
6	51.96		3_OH_,4_αβ_,7,14
7	70.39	7	7,14
8	199.87		
9	135.96		11,16
10	138.46	10	11,16
11	74.53	11	14
12	65.61		2,4_αβ_,13_αβ_,14
13	47.41	13_α_, 13_β_	
14	14.34	14	
15	62.56	15_α_, 15_β_	
16	15.37	16	11,16

a *Measured directly from the spectrum. No statistical error available from fitting. The experimental digital resolution is 0.55 Hz or 0.007*.

**Table 4:** **Experimental and literature ^**13**^C chemical shift data for DON in CDCl_**3**_, acetone-d_**6**_, and DMSO-d_**6**_**.

**^13^C**	**CDCl**_3_		**Acetone-d**_6_		**DMSO-d**_6_	
	**Lit.^a^**	**Exp**.	**Lit.^a^**	**Exp**.	**Lit.^a^**	**Exp**.
2	80.6	80.80	81.6	80.84	80.2	80.62
3	68.6	69.17	69.3	68.60	67.8	68.17^b^
4	43.0	43.21	44.4	43.64	43.7	**44.12**
5	47.2	46.46	46.5	45.79	45.4	**45.75**
6	52.1	51.96	53.0	52.20	51.8	52.18
7	70.2	70.39	70.6	69.71	69.3	**69.61**
8	202.3	199.87	200.7	199.83	200.0	200.58
9	135.7	135.96	135.6	134.68	134.8	135.18
10	138.5	138.4	139.7	138.99	138.2	138.71
11	74.4	74.53	75.4	74.57	74.5	74.84
12	65.6	65.61	66.5	65.66	66.0	**66.31**
13	46.0	47.41	47.5	46.66	46.7	47.11
14	13.9	14.34	14.5	13.71	14.5	**14.84**
15	61.4	62.56	61.5	60.80	60.1	**60.47**
16	14.9	15.37	15.2	14.41	15.1	15.46

a *All literature data obtained from Jarvis et al. (1990)*.

b *Blue indicates increase and red decrease in chemical shift with respect to CDCl_3_. No error estimates available from fitting. The experimental digital resolution is 0.55 Hz or 0.007 ppm*.

The internuclear distances from the NOESY spectra in CDCl_3_ are compared with those from structures modeled with a boat and chair configuration of the B ring (Figures 3A,B). The results are shown in Table 2. The c^2^ between the experimental values and the boat configuration is 17.6 Å^2^, while that with the chair configuration is 6.7 Å^2^, indicating a preference for the chair form. If one were to drop internuclear distances involving any mobile nuclei (i.e., OH, sidechain and CH_3_ signals) from consideration, c^2^ would dramatically decrease to 11.8 Å^2^ for the boat and 2.1 Å^2^ for the chair form. This significantly increases the confidence level in the match of the remaining 18 internuclear distance measurements to the chair form with a standard deviation of 0.35 Å as compared with the boat form at 0.83 Å.

### Full spectral assignment of DON in DMSO-d_6_

**Figure 5:**
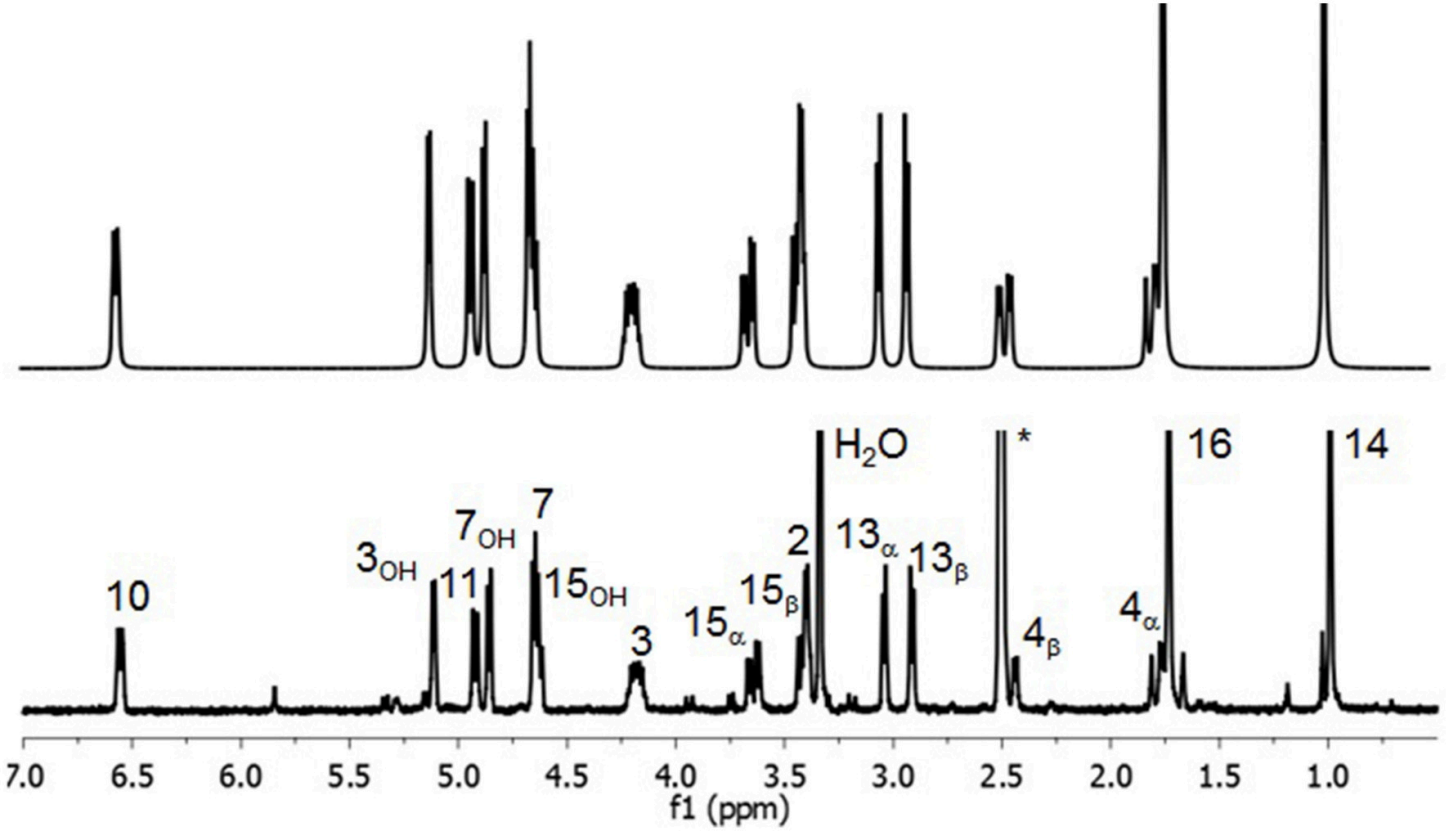
**Experimental (Bottom) and simulated (Top) 300 MHz ^**1**^H spectrum of DON in DMSO-d_**6**_**.

**Figure 6:**
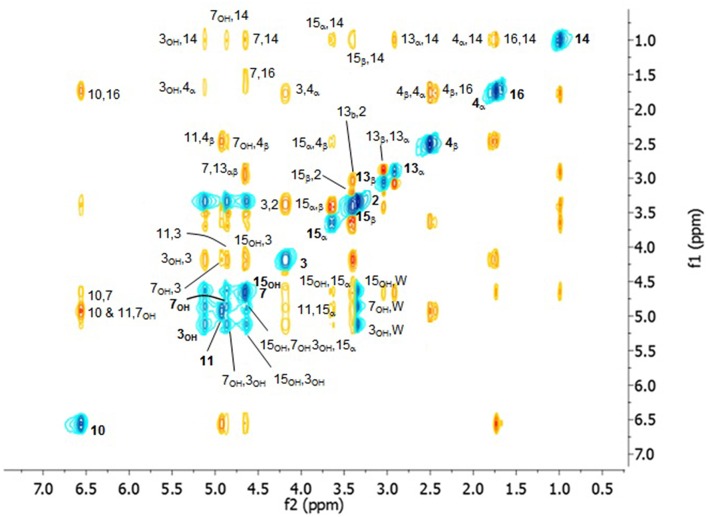
**300 MHz ^**1**^H NOESY of DON in DMSO-d_**6**_ with a mixing time of 1 s**.

The spectrum in DMSO-d_6_ is shown in Figure 5 along with its simulation. Figure 6 shows the corresponding NOESY spectrum. The spectral parameters obtained by simulation and fitting to the experimental spectrum are given in Tables 5, 6 for all five solvents.

**Table 5:** **The chemical shifts (ppm) and associated error (Hz) obtained by fitting the ^**1**^H spectra of DON are in the various solvents**.

		**Chemical Shift (ppm)** ± **Error (Hz)**				
**^1^H**	**CDCl**_3_		**DMSO-d_6_**	**Acetone-d_6_**	**THF-d_4_**	**Methanol-d_4_**
2	3.671 (±0.002)^b^		3.400^c^ (±0.003)	3.499 (±0.004)	3.391 (±0.067)	3.545 (±0.005)
3	4.577 (±0.002)		$\underline{4.180}$ (±0.004)	4.401 (±0.009)	$\underline{4.277}$ (±0.068)	4.378 (±0.004)
3_OH_	2.066 (±0.001)		5.114 (±0.003)	4.270 (±0.004)	4.282 (±0.072)	N.O.
4_α_	2.129 (±0.001)		$\underline{1.770}$ (±0.003)	1.943 (±0.004)	1.861 (±0.065)	1.969 (±0.005)
4_β_	2.247 (±0.002)		$\underline{2.461}$ (±0.003)	$\underline{2.530}$ (±0.004)	$\underline{2.451}$ (±0.074)	$\underline{2.464}$ (±0.004)
7	4.876 (±0.002)		**4.653** (±0.003)	4.833 (±0.009)	4.758 (±0.006)	4.810(±0.005)
7_OH_	3.861 (±0.002)		4.858 (±0.003)	4.041 (±0.006)	4.146 (±0.006)	N.O.
10	6.652 (±0.002)		6.555 (±0.003)	6.612 (±0.007)	6.513 (±0.007)	6.614(±0.004)
11	4.843 (±0.002)		4.922 (±0.003)	5.072 (±0.006)	4.990 (±0.006)	4.959 (±0.004)
13_α_	3.195 (±0.001)		$\underline{2.916}$ (±0.003)	3.056 (±0.014)	2.992 (±0.006)	3.110(±0.005)
13_β_	3.121 (±0.001)		3.041 (±0.003)	3.063 (±0.013)	2.917 (±0.006)	3.073(±0.005)
14	1.170 (±0.001)		0.990 (±0.002)	1.114 (±0.005)	1.061 (±0.005)	1.123 (±0.003)
15_α_	3.932 (±0.002)		3.639 (±0.003)	3.838 (±0.073)	3.702(±0.005)	3.779(±0.005)
15_β_	3.778 (±0.002)		**3.414** (±0.003)	3.728 (±0.055)	3.603(±0.005)	3.697(±0.005)
15_OH_	1.759 (±0.002)		4.631 (±0.003)	3.801 (±0.086)	3.778(±0.005)	N.O.
16	1.930 (±0.001)		1.734 (±0.002)	1.824 (±0.005)	1.776 (±0.005)	1.846 (±0.003)
RMS^a^						

a *Typically 4200 transition were assigned in total. RMS, Root mean square deviation. The largest absolute difference was typically below 0.1 Hz*.

b *The simulations give chemical shifts in Hz units with standard deviation of less than 0.002 to 0.1 Hz. This amounts 5–7 significant figures; therefore, when converting to ppm the shifts should have be recorded from 4 to 6 decimal places. In this case the errors are given in Hz units, as in ppm the error would be 0 up to the third decimal place*.

c *Red indicated increase and blue a decrease in chemical shift with respect to CDCl_3_. The more significant changes are underlined*.

**Table 6:** **Scalar couplings (Hz) and associated error (Hz) obtained by fitting the ^1^H spectra of DON in the various solvents**.

	**CDCl_3_**	**DMSO-d_6_**	**Acetone-d_6_**	**THF-d_4_**	**Methanol-d_4_**
^3^J(2,3)	4.940 (±0.003)	4.121 (±0.006)	4.407 (±0.007)	4.583 (±0.113)	4.428 (±0.008)
^3^J(3,3_OH_)	4.080 (±0.003)	4.112 (±0.006)	4.085 (±0.007)	4.416 (±0.108)	N.O.
^3^J(3,4_α_)	10.980 (±0.003)	11.355 (±0.007)	11.127 (±0.007)	10.986 (±0.126)	11.070 (±0.009)
^3^J(3,4_β_)	4.140 (±0.003)	3.347 (±0.006)	4.368 (±0.007)	4.059 (±0.112)	4.369 (±0.008)
^2^J(4_α_,4_β_)	−14.870 (±0.003)	−14.040 (±0.006)	−14.466 (±0.006)	−14.479 (±0.101)	−14.512 (±0.009)
^3^J(7,7_OH_)	1.940 (±0.003)	3.945 (±0.005)	2.927 (±0.010)	2.647 (±0.008)	N.O.
^3^J(10,11)	5.664 (±0.004)	6.021 (±0.006)	5.911 (±0.009)	6.043 (±0.010)	6.009(±0.006)
^4^J(10,16)	−1.510 (±0.002)	−1.511 (±0.007)	−1.526 (±0.006)	−1.607 (±0.006)	−1.601(±0.007)
^2^J(13_α_,13_β_)	4.220 (±0.003)	4.575 (±0.005)	4.514 (±0.011)	4.565 (±0.010)	5.065 (±0.011)
^2^J(15_α_,15_β_)	−12.032 (±0.004)	−11.230 (±0.006)	−11.961 (±0.120)	−11.600(±0.105)	−11.816(±0.012)
^3^J(15_α_,15_OH_)	5.670 (±0.003)	5.251 (±0.006)	5.696 (±0.138)	5.285(±0.104)	N.O.
^3^J(15_β_,15_OH_)	4.820 (±0.003)	4.739 (±0.005)	4.819 (±0.094)	4.767(±0.107)	N.O.

The assignment of the ^1^H spectrum also departs from previous work concerning the methylene protons at C_4_ and C_15_. The hydroxyl resonances have been previously reported and are consistent with our findings. As in CDCl_3_, 4_α_, and 4_β_are reversed with respect to previous reports (Savard and Blackwell, 1994), where the former has the lower shift, and the vicinal coupling between the 4_α_ and 3 indicates they are eclipsed. In addition, the chemical shift of 4_α_ decreases, while 4_β_ increases significantly between CDCl_3_ and DMSO-d_6_. The assignments of 13_α_ and 13_β_ is the same as in Savard et al. (1987), hence reversed with respect to CDCl_3_, where the shift of 13_α_ decreases sufficiently to be lower than 13_β_ which is less sensitive to the change in solvent environment. The assignment of 13_α_ and 13_β_ were verified by NOESY CP between 13_α_ and 14 and between 13_β_ and 2, respectively. Both 15_α_ and 15_β_ decrease by similar amounts, although their absolute assignment is difficult to verify by NOESY as they both have CP with 14, 11, and 3_OH_. However, 15_α_ is the only one to have a cross-peak with 4_β_.

Other notable trends in ^1^H shifts changes from CDCl_3_ to DMSO-d_6_ are that all the shifts corresponding to the position on the C-ring namely 2, 3, and 4_α_ decrease, and similarly 7, 14, and 16 decrease while 11 increases. Most of the changes in the chemical shifts can be reconciled by the reorientation of 15_OH_ to favor the *trans* rotamer with respect to C_6_, as shown in Figure 3C. The 15_OH_ now situated under the B-ring oriented toward the C-ring side. This increases the distances from 15_OH_ to 4_α_, 7, 14, and 16 hence significantly decreasing their shifts, while decreasing the distances to 4_β_ and 11 hence increasing their shifts.

Again the slow water exchange allows OH shifts to be interpreted. In DMSO-d_6_ the chemical shift of 7_OH_ increases the least by 1 ppm, while 15_OH_ and 3_OH_ increase much more dramatically by approximately 3 ppm each. This can only occur if significant new H-bonding interactions take place involving 15_OH_ and 3_OH_. In response to 15_OH_ being sequestered 7_OH_ is free to interact more strongly with the carbonyl oxygen as indicated by ^3^J_7, 7OH_ changing from 2 to 4 Hz between CDCl_3_ and DMSO-d_6_.

**Figure 7:**
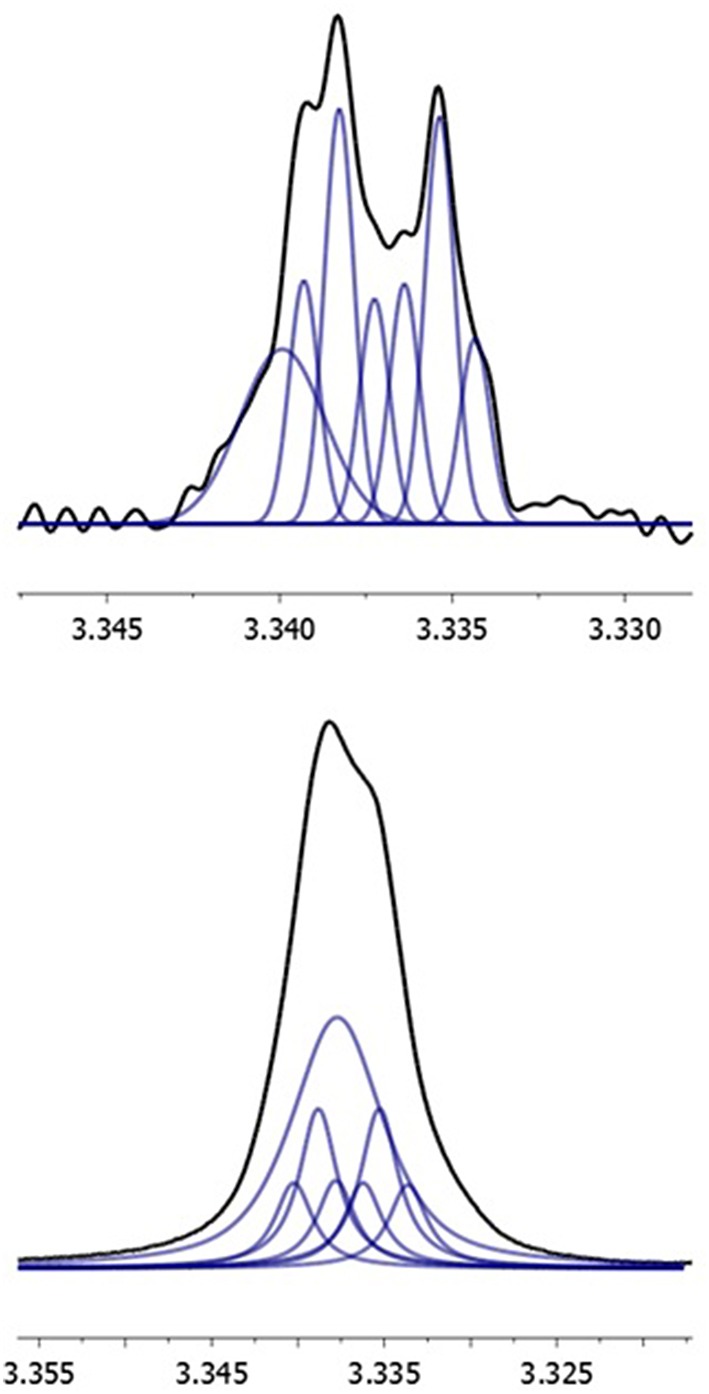
**Expansion of the water signal in the 300 MHz ^**1**^H spectrum of DON in DMSO-d_**6**_**. Left: Gauss-Lorenz apodization with LB = −0.9 Hz and GB = 0.35 Hz. Right: No apodization. Each are deconvolved into a doublet of triplets and one singlet.

Resolution enhancement of the water signal indicates that there are two distinct water signals. One component has a splitting pattern that resembles a doublet of triplets with a linewidth of 0.3 Hz (Figure 7). This is actually an AMNX spin system, where A and X are 15_OH_ and 3_OH_, and the water protons M and N are inequivalent by 0.013 ppm. The coupling constants are ^2^J_MN_ = −7.8 Hz (^2^J_HH_ is estimated from HOD in deuterium exchange study on T2-toxin in CDCl_3_ (Chaudhary et al., 2011), and the outer lines of the MN pattern have 1.3% intensity of inner lines and thus are lost in the noise), ^6^J_AX_ = 0 Hz, and ^2^J_AM_ = ^2^J_NX_ and ^4^J_AN_ = ^4^J_MX_ each ranging from −0.20 to −0.40 Hz. Such a coupling pattern is consistent with an immobile bridging water species, between 3_OH_ and 15_OH_, spanning the mouth of a “binding pocket.” When considering the B- and C-rings together as a seven-membered ring that is bridged by the epoxide, the “binding pocket” is the bottom of the combined ring, opposite to the epoxide bridge.

The other component of the water signal exhibits no fine structure with linewidth of 0.86 Hz. With the water bridged (hence strained) the binding pocket retains essentially the same configuration as in CDCl_3_ where 3 and 4_α_ remain staggered. The exchange rate between water and all three hydroxyl groups is effectively the same near 0.07 to 0.08 s^−1^, which indicates that binding to 15_OH_ and 3_OH_ is stronger in DMSO-d_6_ than in CDCl_3_.

The ^13^C shift changes occurring in DMSO-d_6_ with respect to CDCl_3_ are consistent with the conformation changes proposed, where δ_C5_, δ_C7_, and δ_C15_ decrease when 15_OH_ moves under the binding pocket which also causes δ_C4_, δ_12_, and δ_14_ to increase. The changes in δ_C3_, δ_C4_ and δ_C12_ are also due in part to reorientation of 3_OH_.

Conformational changes were investigated further using NOESY CP in to compare cross relaxation rates *s*_11, 4β_, *s*_15α, 4β_, *s*_13α, 7_, *s*_13β, 7_, and *s*_13β, 2_ between the two solvents. The corresponding internuclear distances r_11, 4β_, r_13β, 2_, r_13α, 7_, and r_13β, 7_ are consistently shorter in DMSO-d_6_ by at least 0.10, 0.15, 0.10, and 0.25 Å. These changes indicate that water binding has caused slight changes in the binding pocket bringing C_4_ and C_3_ closer to C_6_ and C_11_, effectively increasing the degree of folding between the B- and C-rings by decreasing the valence angles at C_2_ and C_5._ As a result, the epoxide ring becomes further removed from the A- and C-rings. This is corroborated by the decrease in chemical shifts of 2, 3, 4_α_, and 7 which would experience deshielding effect of the epoxide ring less strongly. Also the reorientation of 15_OH_ to underneath the binding pocket was confirmed by r_15α, 4β_ decreasing from 2.70 to 2.19 Å.

Additional evidence for changes in the geometry of the B- and C-rings upon water binding is seen in the changes to the scalar couplings in DMSO-d_6_. The dihedral angle between 2 and 3 increases, and between 3 and 4_α_ decrease as indicated by ^3^J_2, 3_ decreasing and ^3^J_3, 4α_ increasing. The valence angle between 4_α_ and 4_β_ increases and that between C_3_ and C_5_ decreases as indicated by ^2^J_4α, 4β_ decreasing. Increased strain in the epoxide ring can be inferred by the increase in ^2^J_13α, 13β_.

### Analysis of the ^1^H spectra of DON in acetone-d_6_, THF-d_4_, and methanol-d_4_

The ^1^H chemical shift trends in the remaining solvents are consistent with the findings in DMSO-d_6_. The shifts of 11 and 4_β_ increase, whereas that of 4_α_ decreases (Table 5). Even though the trends in 7, 2, 3, and 13_α_ are not as extreme as in DMSO-d_6_, they do follow the same sense. Also, in acetone-d_6_ and THF-d_4_ the 3_OH_ and 15_OH_ shifts increase significantly by 2 ppm, which are not quite as large as in DMSO-d_6_. In methanol-d_4_ the hydroxyl signals could not be observed as they were lost to deuterium exchange with the solvent.

Only the water signal in acetone-d_6_ exhibited sufficient resolution such that two components could be found, corresponding to two bound water molecules; however, there were no fine structural features that indicate a bridging binding motif. Cross-relaxation measurements suggest that one of the water molecules was approximately 2.80 Å away from 11.

In all three solvents 15_OH_ does favor the *trans* rotamer; however, not exclusively as in DMSO-d_6_. The remaining mobility in 15_OH_ accounts for the smaller increases in the hydroxyl shifts as well as the weaker trends in 7, 2, 3, and 13_α_. The weaker bonding interaction in the binding pocket for methanol-d_4_ and THF-d_4_ is not just the result of differing solvent polarity as they also contain much more free water, where the water to DON ratio was 14 and 30 to one, respectively. Furthermore, methanol-d_4_ is a hydrogen bonding solvent and therefore would compete with water thereby further weakening the water binding to DON. One would expect to see the same effect in bulk water.

## Discussion

### Water binding in the proposed structure with NOESY in CDCl_3_and DMSO-d_6_

Previous work in our group showed that a water molecule is bound to T-2 toxin at a 1:1 ratio (Chaudhary et al., 2011). The same was observed here for DON in both chemical environments investigated. Water binding to DON was observed through cross-relaxation between water and protons of the binding pocket. Internuclear distances in CDCl_3_ from water to 2, 3, 11, 15_α_ and 15_β_ are 2.99, 2.84, 3.14, 2.93, and 3.22 Å, respectively. This places at least one water molecule beneath the binding pocket in the vicinity of 15_OH_ and 3_OH_. The lack of fine structure in the high resolution water ^1^H signal suggest significant mobility remains in the bound water and thus is unlikely to bridge 3_OH_ to 15_OH_.

In DMSO-d_6_ water binding involves one water molecule bridging the 3_OH_ to 15_OH_. Additional water may be bound elsewhere; however, it would be more mobile in a similar manner to what is observed in CDCl_3_. Unfortunately, no CP between water and 2, 11, or 15_α_ and 15_β_ could be resolved, or found to be adequately free from *t*_1_ noise to obtain reliable distance estimates. It was possible to integrate the cross-peak with 3 giving a distance of *r*_W, 3_ = 2.25 Å which is significantly smaller than in CDCl_3_ and supports the bridging binding mode.

**Scheme 2:**
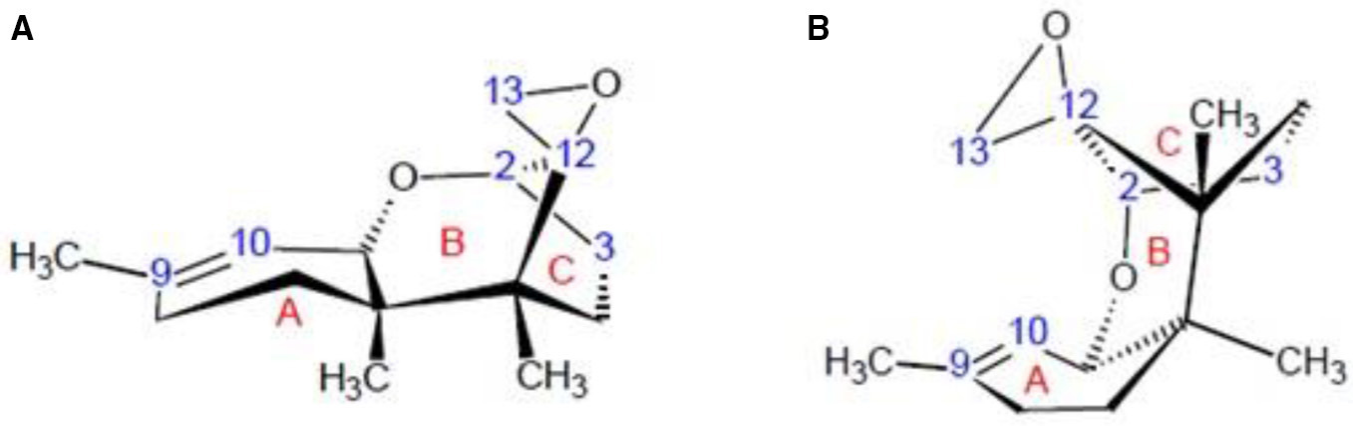
**Three-dimensional stereochemistry of the trichothecene core when (A) the A-ring is in a half-chair, and the B-ring in a chair conformation; and (B) the A-ring is a half-chair, and the B-ring in a boat conformation**.

Considerable conformational variation exists amongst the trichothecenes due to differences in the pucker of the A- and B-rings. X-ray studies indicate a preference for the B-ring to adopt a chair conformation (see Scheme 2) in the crystal phase (Greenhalgh et al., 1984), although boat configurations have been observed for the macrocyclic trichothecenes (Jarvis and Mazzola, 1982; Jarvis and Wang, 1999). Until now, direct evidence for the chair configuration in solution was scarce, apart from some long range scalar coupling interactions observed between 7_β_ and 11 in some systems pointing toward the chair form for the B-ring (Greenhalgh et al., 1989). In the structure presented herein, the B-ring is shown to be in a chair configuration, and the water molecule is bound in the binding pocket in both CDCl_3_ and DMSO-d_6_ (Figure 3).

The major structural differences observed between the two environments occurred with respect to the OH groups, which undergo distinct internal H-bonding and water binding patterns. In CDCl_3_ the 15_OH_ has significant configurational flexibility and undergoes H-bonding with both 7_OH_ and 3_OH_, although 7_OH_ is primarily occupied with H-bonding with the carbonyl oxygen at C_8_. In DMSO-d_6_, the interaction between 15_OH_ and 7_OH_ is lost, and the 15_OH_ interaction with 3_OH_ involves a bridging through a water molecule. This bridged interaction leads to a much stronger binding of the water in DMSO-d_6_ as compared with CDCl_3_. Furthermore, water binding in DMSO-d_6_ leads to slight conformational changes in the B- and C-rings, such that the opening of the combined rings is smaller, thus accommodating the water bridging. Therefore, in non-H-bonding and low polarity environments, in the absence of free water, the configuration of the binding pocket arranges the 3_OH_ and 15_OH_ optimally for donation, hence they will be very reactive to nearby acceptors, such as water, basic amino acid residues, and metal cations.

### Comparison of DON structures in solution with reported structures of bound trichothecenes

The crystal structure of DON and other trichothecenes bound to the yeast ribosome (Garreau De Loubresse et al., 2014) and to *Fusarium* trichothecene acetyltransferases, TRI101 (Garvey et al., 2008) and TRI3 (Garvey et al., 2009), were previously reported. In the bound crystal structures for DON (Garvey et al., 2008; Garreau De Loubresse et al., 2014) and in the solution structure in DMSO-d_6_ described herein, the B-ring is in the chair configuration and the 15_OH_ group is pointing underneath the ring. In DMSO-d_6_, the 15_OH_ is poised underneath the C-ring, whereas when bound in TRI101 it is underneath the A-ring. In the ribosome-bound crystal structure it is unclear whether it is pointing inwards toward the A- or C-ring. By contrast, in CDCl_3_, the 15_OH_ of DON is pointing outwards from the molecule. Interestingly, this is similar to the crystal structures of the ribosome-bound and the TRI101-bound T-2 toxin (Garvey et al., 2008; Garreau De Loubresse et al., 2014), where the 15_OAc_ is unambiguously pointing outwards. Furthermore, a water molecule was observed in the vicinity of the binding pocket in the TRI101-DON and TRI3-15-decalonectrin interactions (Garvey et al., 2008, 2009), in approximately the same distance as observed for DON herein. Furthermore, in the TRI101-DON interact, a strong donor-acceptor interaction was observed between the 3_OH_ and His-156 (Garvey et al., 2008).

Based on these structural insights, we propose the following mechanism of inhibition of protein synthesis. The epoxide ring, known to be essential for toxicity, was not reported to have direct interactions with ribosomal components in the crystal structure presented by Garreau De Loubresse et al. (2014). The specific nature of the epoxide ring making it essential for toxicity is unclear; however, it stands to reason that the epoxide holds the binding pocket in an ideal configuration to bring 3_OH_ and 15_OH_ in close proximity. The NMR structures presented here show that 3_OH_ and 15_OH_ bind to a water molecule in various ways depending on the nature of the solvent. Thus, we hypothesize that in a cellular environment, where free water is limited, the 15_OH_ and 3_OH_ of DON interacts with a magnesium ion in the A-site in the PTC (which is aligned with the normal vector to the plane of the opening of binding pocket) thereby disrupting its activity by changing the local conformation of the PTC. It should be noted that the resolution of the toxin in the ribosome was not sufficient enough to determine whether or not any water was included in the binding. In the event that water is present, it is also possible that the water itself, held in place through the bridging interaction, interacts with the magnesium ion. If the magnesium ion were to be sequestered in either capacity, the local conformation in the PTC would be disrupted; hence, the peptidyl transferase activity would be inhibited.

### Implications of water binding on the toxicity of DON and related trichothecenes

The work presented herein provides new high resolution structural and dynamic information for the trichothecene toxin DON. Our results support previous structural works, but also give new insights with respect to the role of water. Of note, is the detailed hydrogen bonding interaction with water which revealed a bridging between 3_OH_ and 15_OH_. This binding between water and DON is incredibly strong with exchange rates of 0.04–0.12 s^−1^, which represents a Gibbs energy on the order of 77–81 kJ mol^−1^. In a previous study (Chaudhary et al., 2011), we reported water binding with T-2 toxin where the 3_OH_ was also shown to be important; however, in this case, the functional group at C_15_ is substituted with an acetyl group in T-2 toxin, which prevents the formation of the water bridge observed here for DON.

The nature of the water binding is likely very important for toxicity, and the strength of this interaction with the molecule may explain differences in toxicity. For example, our work shows that 3_OH_ plays an important role in water binding, meanwhile substitution at the 3_OH_ in trichothecenes, such as acetylation or glycosylation, has been shown to reduce or eliminate toxicity (Alexander et al., 1999; Poppenberger et al., 2003; McCormick, 2009). It is possible that these modifications at C_3_ increase the number of possible binding modes which are non-optimal for donor-acceptor interactions of the binding pocket with its immediate surroundings, thereby reducing its ability to effectively bind magnesium.

We hypothesize that the role of the epoxide ring in toxicity is in stabilizing the structure of the binding pocket for interactions with water and/or relevant molecules in its vicinity, and that differences in toxicity are related to (a) the reactivity of the binding pocket, which is based on substitution patterns on the trichothecene core, and (b) the orientation of the binding pocket with respect to the magnesium ion in the A-site of the PTC. This hypothesis outlines directions of future inquiries currently under investigation, such as effects of substitutions on water binding, the role of the epoxide ring on water binding, direct donor-acceptor interactions of these toxins with metal centers. The knowledge to be obtained from these studies can be exploited to initiate and/or support the development of detoxification strategies. Strategies to modify the C_3_ have previously been proposed to reduce the impacts of trichothecene contamination of grain using transgenic approaches (Muhitch et al., 2000; Alexander, 2008; Shin et al., 2012). The work herein provides the first insights as to why substitutions at C_3_ reduce toxicity, namely the role of 3_OH_ in water binding. Further analysis of this interaction may lead alternative strategies to disrupt the interaction with water and could therefore be used in trichothecene remediation from contaminated grains.

## Author contributions

NF contributed to roughly half of the writing of this manuscript and brought her perspective as molecular biologist/biochemist to the study to put things into context with FHB research, bringing biological insights, and discussion from the data that was generated. RS was a M.Sc. student working under the co-supervision of PH and FE. She conducted the first round of NMR experiments and provided some ideas for background discussion. DK is a B.Sc. student who was working under the supervision of PH. He conducted the second round of NMR experiments and provided the majority of the data used for this manuscript. PH contributed to roughly half of the writing of this manuscript, provided detailed analysis of the data, prepared the figures and tables. He provided financial contributions to the project and expertise in chemistry and NMR. FE provided the main source of financial contributions to the project, and contributed expertise in FHB.

## Funding

This research was funded by Agriculture and Agri-Food Canada (AAFC) research grants awarded to FE and NSERC grants awarded to PH.

### Conflict of interest statement

The authors declare that the research was conducted in the absence of any commercial or financial relationships that could be construed as a potential conflict of interest.
